# Community Perspectives on Factors Contributing to Resilience for Healthy Aging in Kenya

**DOI:** 10.1002/alz70860_101864

**Published:** 2025-12-23

**Authors:** Linda Norah Khakali, Everline N Onchari, Maureen N Ondire, Catherine N Bikeri, Chinedu T Udeh‐Momoh, Edna N Bosire

**Affiliations:** ^1^ Aga Khan University, NAIROBI, Nairobi, Kenya; ^2^ Aga Khan University, Nairobi, Nairobi, Kenya; ^3^ Female Brain Health and Endocrine Research (FemBER) Consortium, Newcastle, Edinburgh, London, United Kingdom; ^4^ Wake Forest University School of Medicine, Winston‐Salem, NC, USA; ^5^ Aga Khan University Brain and Mind Institute, Nairobi, Nairobi, Kenya; ^6^ Brain and Mind Institute, Aga Khan University, Nairobi, Nairobi, Kenya

## Abstract

**Background:**

The global elderly population is increasing and expected to double in the next decade. Aging increases the likelihood of adversities, with resilience becoming an important aspect of healthy aging. Understanding how individuals conceptualize resilience offers an opportunity to create contextually relevant interventions for promoting healthy aging. This study aimed to explore adults’ perspectives on factors influencing resilience in Kenya.

**Method:**

Using qualitative methods, 36 adults aged 35 years and above were purposively recruited from various communities in Nairobi County to participate in interviews. We asked questions about their understanding of aging, adversity and resiliency. We also provided them with case scenarios of two individuals responding differently to the same adversity and asked them to discuss what influenced one to respond better when compared to the other. Interviews were audio‐recorded, transcribed verbatim and thematically analyzed.

**Result:**

Participants understood resilience as the ability to bounce back after adversity. They highlighted that adversities could accelerate physical and cognitive aging, manifesting as signs of faster aging, increased risk of illnesses, cognitive decline, and other comorbidities of unhealthy aging. Reflecting on the cases, they narrated that resilience was promoted by internal factors (inner strength, mindset, routine practices, and attitude) and external factors such as social support from family and friends; psychological support including professional interventions and positive reinforcement from loved ones and medical interventions. Physical well‐being was also linked to health awareness and proactive activities. Internal factors were seen as adaptable and shaped by the individual's environment and experiences.

**Conclusion:**

We argue that resilience, key to healthy aging, is shaped by both internal factors and external factors. These factors should be considered when designing interventions aimed to promote healthy aging in resource constrained settings.

